# Guideline-directed medical therapy in heart failure patients: impact of focused care provided by a heart failure clinic in comparison to general cardiology out-patient department

**DOI:** 10.1186/s43044-020-00088-8

**Published:** 2020-08-24

**Authors:** Jeeva Joseph, Stephy P S, Jude James, Suja Abraham, Jabir Abdullakutty

**Affiliations:** 1 Department of Pharmacy Practice, Nirmala College of Pharmacy, Muvattupuzha, Ernakulam Dist, Kerala, 686661 India; 2 Department of Cardiology, Lisie Hospital, Kochi, Kerala India

**Keywords:** Heart failure clinic, Guideline-directed medical therapy, Target dose, Personalized care

## Abstract

**Background:**

The usage of guideline-directed medical therapy (GDMT) in the treatment of heart failure (HF) has shown to reduce morbidity and mortality. However, majority of the HF patients do not receive GDMT or do not achieve the target dose. Literature has shown that the patients who are managed in HF clinics receive GDMT and target doses of disease-modifying drugs (DMD) when compared to those treated in other general cardiology outpatient departments (OPD’s). It was a retrospective hospital-based study in which patients treated in HF clinic and other cardiology OPD in the year of 2017 were included (200 patients in each arm). The aim of this study was to assess the impact of heart failure clinics in medication therapy management including usage of guideline-directed medical therapy, if target dose specified by the guideline is achieved and time to reach target dose in comparison to other general cardiology OPD’s. IRB and IEC approval were obtained before the commencement of the study. Data relevant to the study were obtained from the electronic medical record (EMR) and were compared between the study groups to see for the adherence to guideline and achievement of target doses. Data storage and analysis were performed using SPSS Version 24. A significance level of 5% was used.

**Results:**

The usage of GDMT was higher in HF clinic when compared to other cardiology OPD (81% vs 55%, *P* = 0.001). A significantly higher number of patients in HF clinic achieved target dose when compared to other cardiology OPD (58% vs 29% -betablockers, 45% vs 9% -ACEI/ARB/ARNI, *P* = 0.000). Moreover, the number of eligible patients receiving DMD was found to be higher in HF clinic (98% vs 85% -betablockers, 69% vs 44% -ACEI/ARB/ARNI, 76% vs 44% -MRA). Also, the patients in HF clinic attained the target doses faster when compared to other cardiology OPD. In addition, there was better improvement in ejection fraction, as well as decreased rate of rehospitalisation and mortality in patients managed in HF clinic.

**Conclusion:**

HF clinics were compared with other cardiology OPD for various parameters and it was observed that HF clinics were better than other cardiology OPD in maintaining the medication therapy management.

## Background

In patients with heart failure (HF), the goals of treatment are to improve their clinical condition, functional capacity, quality of life, and to prevent the events of hospital readmissions and mortality. The guidelines of various organizations such as American College of Cardiology (ACC), American Heart Association (AHA), Heart Failure Society of America (HFSA), and ESC (European Society of Cardiology) provide a clear idea on the drug choice, drug dose, and target dose to be achieved in heart failure patients and recommend that patients with heart failure with reduced ejection fraction (HFrEF) be treated with maximum tolerated doses of appropriate neurohormonal blockers unless contraindicated or not tolerated [1–3]. It is now recognized that usage of guideline-directed medication therapy (GDMT) helps in reducing HF hospitalization, mortality, and improving functional capacity [4]. Despite immense positive evidence [5–12], < 25% of patients with HFrEF are on the appropriate target doses of medical therapy [13].

The optimization of GDMT is primarily carried out by a cardiologist or primary care provider in the outpatient setting typically resulting in delayed optimization due to relatively infrequent patient visits and laboratory monitoring [14–16]. Several studies have evaluated the efficiency of other healthcare professional-led clinics like the nurse-led titration clinics which demonstrated increased utilization rates of GDMT and an improved proportion of patients on target doses [17–20]. However, the impact of a heart failure clinic in the usage of guideline-directed medical therapy has not been studied in an Indian setting. The aim of this study was to assess the impact of focused healthcare provided by a heart failure clinic in the usage of GDMT in an Indian setting by comparing the patients approaching heart failure clinic (HF clinic) and other cardiology outpatient department (OPD).

## Methods

The study was a retrospective hospital-based study conducted in a tertiary care hospital in southern India in which patients with HF who consulted either in the HF clinic or other general cardiology OPD during the year 2017 (200 patients in each arm) were enrolled. Institutional Review Board (IRB) and Institutional Ethics Committee (IEC) (Reg no: ECR/40/inst/KL/2013/RR-16) approval were obtained before the commencement of the study.

### Guideline-directed medical therapy

The patients with heart failure were managed based on the recommendation by the ESC guidelines. As per ESC guideline 2016, when patients were diagnosed with HF and are symptomatic with EF < 35%, they should be initiated with angiotensin converting enzyme inhibitors (ACEI)/angiotensin receptor blockers (ARB) and beta blocker (BB). If the patients still remain symptomatic, mineralocorticoid receptor antagonist (MRA) should be added to the regimen. If the LVEF remains < 35% with persistence of symptoms, guideline recommends using angiotensin receptor neprilysin inhibitor (ARNI) instead of ACEI/ARB. Cardiac Resynchronisation Therapy (CRT) should be considered the treatment of choice when the patient is on sinus rhythm and QRS duration ≥ 130 ms. Ivabradine should be initiated when the patient is on sinus rhythm with heart rate > 70 bpm. Still if the patient had resistant symptoms digoxin or hydralazine and isosorbide dinitrate (H-ISDN) or left ventricular assist device (LVAD) or heart transplant should be considered. Based on these recommendations each HF patient was evaluated for the use of guideline-directed medical therapy.

### Data collection and analysis

Patient data relevant to the study was obtained from the electronic medical record (EMR) during three time points, i.e., first month, sixth month, and twelfth month after the first visit, and the collected data were then evaluated between the study groups to assess the usage of GDMT. Attainment of evidence-based target doses of disease-modifying drugs (ACEI’s, ARB’s, beta-blockers, and MRA’s) and time to reach target dose were also evaluated. The patients were categorized to four groups based on the percentage of target dose achieved as group 1 (0–25%), group 2 (26–50%), group 3 (51–75%), and group 4 (76–100%). Data storage and analysis were performed using Microsoft Excel 2010 and SPSS Version 24. For the comparison of continuous variable, we used independent sample *t* test and paired *t* test, and the categorical variables were compared using *χ*^2^-likelihood ratio test. All the *p* values were two-tailed, and a significance level of 5% was used.

## Results

Demographic details such as age, gender, social habits, risk factors, and comorbidities are presented in Table 1 and are comparable among the two study groups. We could see a male predominance in both the groups, and mean age of the patients was 60.64 ± 11.44 years in HF clinic and 63.62 ± 10.48 years in other cardiology OPD. The most prevalent risk factor was diabetes mellitus (60% HF clinic vs 65% in other cardiology OPD) and the most reported comorbidity was anterior wall myocardial infarction (AWMI) among the 2 study groups (42% in both study groups). Among their social habits, more smokers were reported in HF clinic whereas equal number of alcoholics in both study groups.

**Table 1:** Demographic characteristics

**Demographics**		**% of patients in HF clinic**	**% of patients in other cardiology OPD**	***P*** **value**
**Male gender**		80%	73%	0.24
**Smoking**	Ex-smokers	48%	42%	0.67
	Current	8%	11%	
**Alcohol drinking**^**a**^		59%	59%	1.00
**Age**	30–45 years	9%	8%	0.24
	46–60 years	43%	25%	
	61–75 years	32%	59%	
	76–90 years	11%	8%	
		**Number of patients**	**Number of patients**	
**Risk factors**				
**Hypertension**		84 (42%)	82 (41%)	0.88
**Diabetes mellitus**		120 (60%)	130 (65%)	0.46
**Dyslipidemia**		54 (27%)	42 (21%)	0.32
**Hypothyroidism**		14 (7%)	12 (6%)	0.77
**Hyperthyroidism**		8 (4%)	4 (2%)	0.40
**Comorbidities**				
**Coronary artery disease**		70 (35%)	64 (32%)	0.65
**Angina**		14 (7%)	10 (5%))	0.55
**Anterior wall myocardial infarction**		84 (42%)	84 (42%)	1.00
**Atrial fibrillation**		16 (8%)	20 (10%)	0.62
**Cardiomyopathy**		38 (19%)	30 (15%)	0.45
**Stroke**		6 (3%)	4 (2%)	0.65
**Chronic liver disease**		8 (4%)	2 (%)	0.17
**Chronic kidney disease**		20 (10%)	32 (16%)	0.20

The demographic details and their comparison between the study groups

^a^Alcohol drinking in the study is defined as those who consumed alcohol occasionally. No binge and excessive alcohol consumers were observed in the study population

### Guideline-directed medical therapy

The use of guideline-directed medical therapy between patients visiting heart failure clinic and other cardiology OPD was compared using the chi-square test and found that 81% of the patients in the HF clinic received GDMT while only 55% in the other cardiology OPD received, and the difference was statistically significant (*P* = 0.001). Among those who received GDMT, 59.6% were from HF clinic whereas 40.4% were from the other study group. Compared to patients consulting other cardiac OPDs, HF clinic patients were more likely to receive GDMT. Among the 81% of patients receiving GDMT in HF clinic, 53.08% of patients were less than the ages of 60 and 76.25% of patients were males, and among 55% patients receiving GDMT in other cardiology OPDs, 41.81% were less than the age of 60 years and 69.09% were males.

### Eligibility and GDMT

**Fig. 1:**
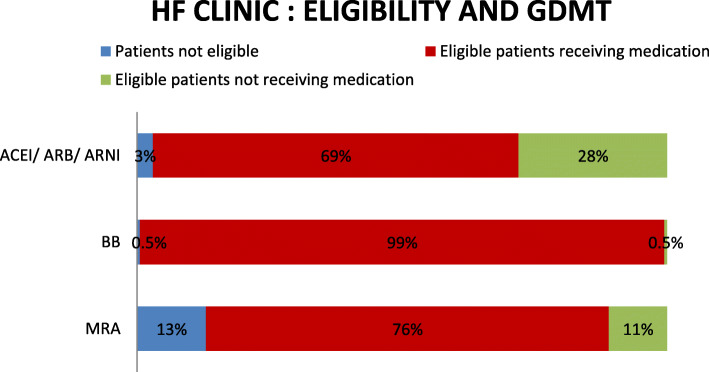
Eligibility and provision of GDMT in HF clinics. Figure 1 presents the eligibility of patients and the extend of usage of GDMT in HF clinic. Here, the patients are divided into three categories that is patients eligible to receive GDMT and had received, patients eligible to receive GDMT and had not received, and those not eligible to receive the GDMT

**Fig. 2:**
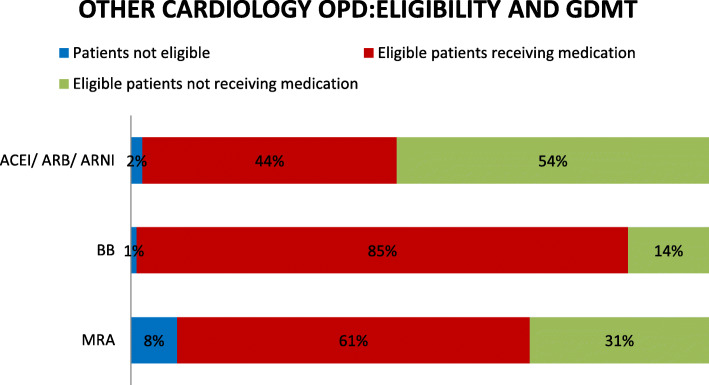
Eligibility and provision of GDMT in other general cardiology OPD. Figure 2 presents the eligibility of patients and the extend of usage of GDMT in other general cardiology OPD. Here, the patients are divided into three categories that is patients eligible to receive GDMT and had received, patients eligible to receive GDMT and had not received, and those not eligible to receive the GDMT

ACEI/ARB/ARNI, β blockers, and MRA are the major therapeutic options for heart failure management as per ESC guideline. In both the study group, number of patients with HF eligible for each therapy is shown in Figs. 1 and 2. Most of the patients were eligible for beta blocker therapy followed by ACEI/ARB/ARNI and few for MRA in both study groups. We analyzed the number of eligible patients receiving GDMT and found that 99% of eligible patients received beta blockers in HF clinics and 85% in other cardiology OPD, 69% received ACEI/ARB/ARNI in HF clinic while only 44% in other study group, and 76% received MRA in HF clinics but only 61% in other cardiology OPD. Patients who were found not eligible to receive the drug were those who could not tolerate the drug or were contraindicated.

### Combination of drugs

As per ESC guidelines, use of triple drug therapy is the primary treatment option for heart failure. About 80% of patients in HF clinic and 53% patients in other cardiology OPD received triple drug therapy. The commonly used drug combination was BB + ARB + MRA (57% in HF clinic vs 26% in other cardiology OPD) followed by BB + ACEI + MRA (15% in HF clinic vs 17% in other cardiology OPD), and BB + ARNI + MRA (15% in HF clinic vs 10% in other cardiology OPD). Dual therapy was followed in 20% patients in HF clinic and 47% patients in other cardiology OPD. The commonly used dual drug combinations were BB + MRA, ACEI + MRA, ARB + MRA, ARNI + MRA, BB + ACEI and among which the mostly used was an ARB + MRA (19% in other cardiology OPD vs 7% in HF clinic).

### Target dose achievement

As Table 2 shows, the percentage of patients who attained their target doses in both study groups during the first, sixth, and twelfth months of the study period were evaluated. We compared the data between the two study groups and it was seen that within the first month greater percentage of patients managed in HF clinic attained target doses of beta blockers and ACEI/ARB/ARNI, and the difference was statistically significant. The same trend continued through sixth to twelfth month. Even though, statistically significant difference was not observed in the proportion of patients taking MRA between the study groups, a greater percentage of patients in HF clinic attained target doses within first and sixth month when compared to patients in other cardiology OPD**.**

**Table 2:** Comparison of target dose achievement in HF clinic and other cardiology OPD

Drugs	Study group	1st month	*P* value	6th month	*P* value	12th month	*P* value
		No. of patients		No. of patients		No. of patients	
BB	HF clinic (*n* = 196)	32 (16.32%)	0.06	76 (38.77%)	0.04	116 (59.18%)	0.0001
	Other cardiology OPD (*n* = 170)	16 (9.41%)		46 (27.05%)		58 (34.11%)	
ACEI/ARB/ARNI	HF clinic (*n* = 138)	12 (8.69%)	0.04	66 (47.82%)	0.0001	90 (65.21%)	0.0001
	Other cardiology OPD (*n* = 88)	2 (2.27%)		12 (13.63%)		18 (20.45%)	
MRA	HF clinic (*n* = 152)	140 (92.10%)	0.34	144 (94.73%)	0.35	144 (94.73%)	0.46
	Other cardiology OPD (*n* = 122)	110 (90.16%)		112 (91.80%)		116 (95%)	

The attainment of target doses of the disease modifying drugs and their comparison between the study groups

*BB* beta blockers, *ACEI* angiotensin converting enzyme inhibitors, *ARB* angiotensin receptor blockers, *ARNI* angiotensin receptor neprilysin inhibitor, *MRA* mineralocorticoid receptor antagonist

### Percentage of target dose of disease-modifying drugs attained

The percentage of target dose the disease-modifying drugs achieved were analyzed at the end of 12th month from the baseline, and the results are shown in Table 3. Among beta blockers, commonly used agents were metoprolol, bisoprolol, nebivolol, and carvedilol. Out of these agents, metoprolol was the widely used drug while bisoprolol attained the target dose at the earliest. Comparatively a greater number of patients in HF clinics attained the greater percentage of target dose of beta blockers during the study period.

**Table 3:** Percentage of target doses achieved by the drugs

Category	HF clinic		Other cardiology OPD	
	Baseline	At 12 months	Baseline	At 12 months
Beta blockers				
0–25%	55%	17%	23%	17%
26–50%	27%	36%	52%	36%
51–75%	8%	26%	19%	36%
76–100%	10%	21%	6%	11%
ACEI/ARB				
0–25%	55%	30%	52%	27%
26–50%	26%	21%	28%	33%
51–75%	5%	5%	12%	23%
76–100%	14%	44%	8%	17%
MRA				
0–25%	-	-	-	-
26–50%	25%	2%	38%	20%
51–75%	-	-	-	-
76–100%	75%	98%	62%	80%

The percentage of target doses achieved by the patients in each study group by the end of the twelfth month. Here the patients have been divided into four categories based on the percentage of target dose attained as: 0–25%, 26–50%, 51–75%, 76–100%

Generally, ARBs/ARNIs were preferred over ACEIs in the hospital, and the only used ACEI was found to be Ramipril (only 14.5% received Ramipril), and target dose of Ramipril was attained in greater number of patients in HF clinic when compared to other cardiology OPD. Losartan, telmisartan, and valsartan were the most used ARBs, and among these, use of losartan was predominant but target dose of valsartan was achieved at the fastest. Number of patients attaining target doses of ARBs and ARNI was higher in HF clinic when compared to other cardiology OPD. Spironolactone was the mostly used MRA followed by eplerenone. Patients on spironolactone attained target dose at a rate higher than eplerenone.

### Time to reach target dose

**Fig. 3:**
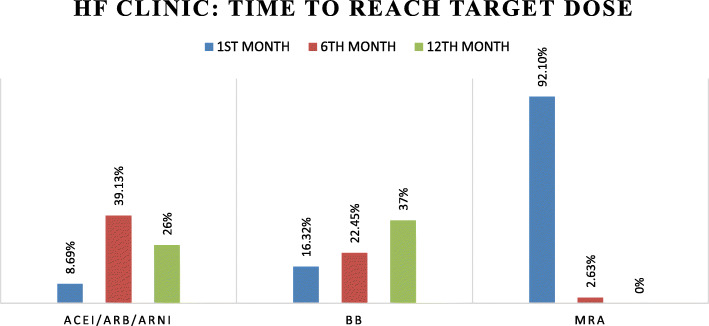
Time to achieve target drug dose in HF clinic. Figure 3 presents the time taken by the patients to reach the target dose of the disease modifying drugs in the HF clinic

**Fig. 4:**
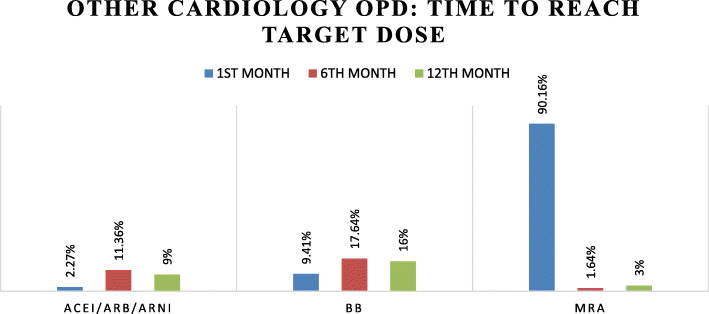
Time to achieve target drug dose in other general cardiology OPD. Figure 4 presents the time taken by the patients to reach the target dose of the disease-modifying drugs in the other general cardiology OPD

As shown in Figs. 3 and 4, we analyzed how much time each patient took to reach the target dose in both study groups. In case of MRA, more than 90% of patients achieved target dose within 1 month. This was because the initial dose of MRA was same as its target dose. For ACEI/ARB/ARNI, 8.69%, 39.13%, 26%, and 2.27%, 11.36%, 9% of patients correspondingly from HF clinic and other cardiology OPD took 1, 6, and 12 months to reach target dose. While for attaining target doses of beta blockers 16.32%, 22.4%, and 37% of patients in HF clinic and 9.41%, 17.64%, and 16% of patients in other cardiology OPD respectively took 1, 6, and 12 months to achieve the target dose of BB.

### Impact of usage of GDMT

Clinical improvement was reflected as there was statistically significant improvement in EF from 28.12 during first month to 38.59 by the end of twelfth month in patients managed in HF clinic (*P* = 0.001) while there was no significant improvement in EF of patients in other general cardiology OPD (33.87 in first month and 34.03 in twelfth month, *P* = 0.38). Moreover, at the end of 1 year, there was significant difference in the number of events of rehospitalisation (65 in HF clinic vs 189 in other cardiology OPD, *P* = 0.000) and mortality (2% in HF clinic vs 8% in other cardiology OPD, *P* = 0.05). All these might be attributed to the increased usage and adherence to the guideline in the patients treated in HF clinic.

## Discussion

From 2008 through 2014, among the three top diagnoses targeted by the Hospital Readmissions Reduction Program (HRRP), HF had the highest number of hospitalizations, and also reported that HF had the highest rate of 30-day readmissions at 23.5% [21]. Hence, heart failure can be regarded as a condition that requires focussed care and management based on the patient clinical and laboratory monitoring. There was a fall in the mortality rate from 57 (1979–1984) to 48% (1996–2000) in HF patients demonstrating the improvement in survival after the diagnosis of heart failure [22]. This improvement in the survival rate is contributed by the development of newer treatment strategies [2]. Large randomized clinical trials conducted have shown the benefit of using ACEIs, ARBs, ARNI, BB, and MRA in reducing the mortality rate and rehospitalisation [2, 23]. The guidelines also strongly recommend the use of combination of all these agents (ACEI/ARB/ARNI + BB + MRA) in all heart failure patients [2]. Despite well-validated guidelines, there continues to be an underutilization of appropriate GDMT in patients with HF. When compared to usual care, specialized clinics for the up-titration and maintenance of BB, ACEI, and ARB dosing in the heart failure population has shown superior performance [17–20, 24]. There is a suggestion within the literature that speciality clinics led titration of neuro-hormonal blocking agents in patients with heart failure result in fewer hospital admissions and improved mortality [4, 18, 25].

In our study a statistically significant difference in the usage of guideline-directed therapy between the study groups is evident and could be due to more focused care provided by the heart failure clinic. Heart failure clinics which are widely practiced in other countries have shown to reduce the events of rehospitalization, mortality, and also have shown to improve the patient outcomes by strict adherence to GDMT, and this acted as the basis for introduction of heart failure clinics into Indian healthcare setting. When compared to the other cardiology OPD, more time is devoted for HF patients to provide personalized care which resulted in timely follow-up and alteration of the therapy based on individual patient needs as specified in the guideline. As little is known about the usage of GDMT in India, Practice Innovation and Clinical Excellence India Quality Improvement Program (PIQPIA) registry was started in India (supported by American College of Cardiology Foundation) in 10 centers to record the use of GDMT and stated that about two-third of the patients (EF < 40%) did not have the documented receipt of GDMT. Hence the focused care programs like HF clinics could help in the improved adherence to GDMT [26]. The results of PIQPIA showed that the GDMT was higher in patients of age greater than 65 years and in women, but in this study, the GDMT was given more in female and those below 60 years of age.

In a contemporary registry in the USA, the study result showed that a large proportion of the eligible patients did not get the target doses of the drug and multiple factors were associated independently with this, hence, emphasize the importance of quality improvement processes in achieving the target doses [27]. Heart failure clinics are associated with the increased use of target doses of the drug when compared to other cardiology OPDs and can be seen as a quality improvement process. The proportion of patients receiving the target doses of BB and ACEI/ARB/ARNI was higher in patients approaching the heart failure clinics during the sixth and twelfth month when compared to those in other cardiology OPDs. In spite of the 20-year evidence and the latest updates of the treatment guidelines, the adherence of the physicians to the guideline-directed medical therapy is still low; there remains a significant gap in its usage on a population level [28]. Hence the specialized management programs like the heart failure clinics recognize and address the various barriers to the guideline adherence and hence can improve the outcome of heart failure patients.

The implementation of specialized care clinics can ensure that the patients receive optimal, individualized, safe, and effective drug therapy. The concept of specialized clinics is well established in foreign countries which have shown to have improved patient outcomes, namely anticoagulation clinic, asthma clinic, diabetes clinic, rheumatology clinic, and immunization clinic. In an Indian setting, the adoption of anticoagulation clinics can be set forth as the best example to demonstrate the impact of specialized clinic in improving patient health outcomes. Hence, the introduction of such specialized clinics to other healthcare departments in a country like India could possibly reduce the health economic burden and enhance patient overall outcomes.

## Conclusion

In this study, we observed that in patients treated under HF clinic the adherence to guideline-recommended pharmacotherapy was high. Drug doses were titrated periodically in patients in HF clinic on the basis of their cardiac function and laboratory parameters. Significantly higher number of patients in HF clinic attained target dose of drugs within the study period. Provision on personalized care through HF clinic ensured the eligible patients received GDMT and had produced a significant impact in HF patients. Hence the study concluded that HF clinics are better and more efficient than usual care given through other cardiology OPD.

## Data Availability

It will be made available later as asked.

## Abbreviations

HF: Heart failure
EF: Ejection fraction
ESC: European Society of Cardiology
GDMT: Guideline-directed medical therapy
DMD: Disease-modifying drugs
HFrEF: Heart failure with reduced ejection fraction
OPD: Out-patient department
ACEIs: Angiotensin converting enzyme inhibitors
MRAs: Mineralocorticoid receptor antagonist
BB: Beta blockers
ARB: Angiotensin II receptor blockers
ARNI: Angiotensin receptor neprilysin inhibitors
EMR: Electronic medical record
AWMI: Anterior wall myocardial infarction
